# Aspiration pneumonia in head and neck cancer patients undergoing concurrent chemoradiation from India: Findings from a post hoc analysis of a phase 3 study

**DOI:** 10.1002/cam4.4210

**Published:** 2021-09-08

**Authors:** Vijay Patil, Vanita Noronha, Sameer Shrirangwar, Nandini Menon, George Abraham, Arun Chandrasekharan, Kumar Prabhash

**Affiliations:** ^1^ Department of Medical Oncology Tata Memorial Centre HBNI Mumbai India

**Keywords:** adverse event, aspiration pneumonia, chemoradiation, complication, head and neck cancer

## Abstract

**Background:**

There are limited data from low‐ to middle‐income countries (LMIC) on the incidence, risk factors, treatment outcomes, and antibiotic susceptibility spectrum of aspiration pneumonia (AsP).

**Methods:**

We conducted a post hoc analysis of a randomized control trial in which adult patients with locally advanced head and neck cancers had received 66–70 Gy of radiation combined with cisplatin 30 mg/m^2^ weekly for 6–7 weeks or cisplatin at the same dose with nimotuzumab 200 mg once weekly till the completion of radiation. The following data were extracted and analyzed—the incidence of AsP, time to the onset of AsP, risk factors, treatment outcomes of AsP, and its impact on progression‐free survival (PFS), locoregional control (LRC) rates, and overall survival (OS).

**Results:**

Out of 536 patients enrolled in the study, 151 (28.3%, 95% confidence interval [CI] 24.5–2.1) patients developed AsP. The median time to develop AsP was 39 days (95% CI 34–44). Only baseline dysphagia (odds ratio = 3.76, 95% CI 1.05–13.51, *p* = 0.042) was associated with a significant risk of development of AsP. Among the patients in which pathogenic organism was isolated (69 patients), gram‐negative species was isolated in 63 patients (89%). Cisplatin at 200 mg/m^2^ or more was delivered in 312 (81%) patients in the non‐AsP cohort versus 107 (70.9%) patients in AsP cohort (*p* = 0.014). There was no statistical difference in LRC (hazard ratio [HR] = 1.057; 95% CI 0.771–1.448), PFS (HR = 1.176; 95% CI 0.89–1.553), and OS (HR = 1.233; 95% CI 0.939–1.618) between the two cohorts.

**Conclusion:**

Aspiration pneumonia is a common complication in head and neck malignancies and patients with baseline dysphagia are at high risk. Gram‐negative bacteria are the predominant causative agents. The use of broad‐spectrum antibiotics results in resolution of symptoms.

## Graphical abstract

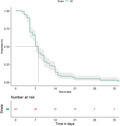

## 1 INTRODUCTION

Head and neck cancer is one of the commonest cancers in the Indian subcontinent.^1^ Most of these patients (>80%) present in the locally advanced stage^2^ and as a result, a substantial proportion of these patients receive concurrent chemoradiation (CTRT). Acute adverse events, especially aspiration pneumonia (AsP) is a worrisome complication of CTRT.^3^ AsP is a common cause of morbidity and mortality^4^, ^5^, ^6^, ^7^ and is probably the most important factor contributing to non‐cancer and unknown deaths.^8^ In a series from a premier tertiary care center in India, it was shown that AsP contributed to 60% of in‐hospital deaths in head and neck cancer patients.^4^ In addition, AsP developing during therapy, leads to gaps in radiation and concurrent chemotherapy,^3^ leading to poorer oncological outcomes.

The incidence of AsP reported in the literature ranges from 5.3% to 46.8%^6^, ^7^, ^9^, ^10^, ^11^, ^12^ and correlates with the incidence of mucositis.^13^ The potentially lethal nature of AsP, with mortality rates of up to 32.5%^7^ has led to the adoption of practices like use of cisplatin in the Indian subcontinent which is associated with a lower rate of mucositis and neutropenia. Administration of weekly cisplatin (30–40 mg/m^2^) serves as a better radiosensitizer over other chemotherapeutic agents like carboplatin.^14^ Heterogeneity in its definition, diagnostic modalities, limited data on the pathogenic microbiological flora from low‐ to middle‐income countries (LMIC), its impact on cancer‐directed treatment and oncological outcomes, have led to limited progress in the field of AsP occurring during chemoradiation. Understanding the microbial flora for AsP may have a significant impact on the antibiotic regimens and outcomes of patients with AsP. To address this lacuna, we performed this analysis with the key objectives of estimating the incidence, risk factors, antibiotic susceptibility spectrum, treatment outcomes of AsP, and its impact on oncology outcomes.

## 2 METHODS

### 2.1 Study details

This was a post hoc analysis of a previously published randomized control trial of cisplatin and nimotuzumab versus cisplatin alone radical CTRT in a locally advanced head and neck squamous cell carcinoma.^15^ The study was conducted in patients undergoing concurrent CTRT in Solid Tumors Unit 2 of Tata Memorial Centre, Mumbai from 2012 to 2018. Adult patients with locally advanced head and neck cancers who were fit at baseline for radical chemoradiation were included in the study. The decision for CTRT was decided in a multidisciplinary tumor board after clinical evaluation, laboratory reports including complete blood count, renal function tests, liver function tests, electrocardiogram and two‐dimensional echocardiogram, pure tone audiometry to assess fitness for cisplatin, and contrast‐enhanced computerized tomography scan image of the head, neck, and thorax for staging. All patients underwent speech–swallow, dental, and nutritional assessment prior to the start of therapy. The patients received radiotherapy (RT) combined with cisplatin 30 mg/m^2^ weekly for 6–7 weeks or cisplatin at the same dose with nimotuzumab 200 mg once weekly till the completion of RT. The dose received was between 60–70 Gy and 1.8–2 Gy per fraction, 5 days a week. Patient received weekly cisplatin or weekly cisplatin and nimotuzumab depending on the arm. They were assessed weekly during the therapy and were followed up at regular intervals till death.

### 2.2 Variables

The primary endpoint of the study was progression‐free survival (PFS). The key secondary endpoints were locoregional control (LRC), overall survival (OS), and adverse events. The study was approved by the Institutional Ethics Committee and was conducted in accordance with the ethics guidelines issued by the Declaration of Helsinki and good clinical practice guidelines. The study was registered with the Clinical Trial Registry of India (trial registration identifier CTRI/2014/09/004980).

### 2.3 Data collection

From the database of this randomized control trial, the following data were extracted—demographic details, tumor site and staging details, treatment compliance, the incidence of AsP during and after chemoradiation (180 days post‐completion), time to the onset of AsP from the start of chemoradiation, the delays in chemotherapy, and RT due to AsP (PFS, LRC rates, and OS).

### 2.4 Endpoint definition

Aspiration pneumonia was defined subject to the fulfillment of the following three criteria.^3^ First, the presence of at least two of the following symptoms—fever, cough, or breathlessness. Second, the presence of features of pneumonia on imaging or clinical examination like crepitations or bronchial breathing. Third, the presence of aspiration suspected either clinically (on history) or on video fluoroscopic imaging. The time to develop AsP was the duration between the start of chemoradiation to the diagnosis of AsP. The time to resolution was the time from diagnosis of AsP to the date of the resolution of clinical symptoms which were lasting for >72 h. PFS was defined as the duration from the date of randomization to the date of progression according to Response Evaluation Criteria in Solid Tumors [RECIST], version 1.1. LRC was defined as the time between the date of randomization and the date of locoregional failure. OS was calculated as the time from the date of randomization to the date of death.

### 2.5 Statistical analysis

SPSS version 20 and RStudio 3.6.2 were used for analysis. Descriptive statistics were performed. Continuous and non‐continuous data were compared using Mood’s median and Fisher’s exact test, respectively. The crude incidence of AsP was estimated with 95% confidence interval (CI) calculated using the Agresti–Coull method. The incidence of AsP was also estimated using competing risk analysis. The median time with 95% CI to the development of AsP, time to resolution of the AsP, PFS, LRC, and OS were estimated using the Kaplan–Meier method. Brookmeyer and Crowley method was used for the construction of the 95% CI. Univariate analysis of factors predicting the development of AsP and affecting its resolution on first‐line antibiotics was performed using the fisher's test. Binary logistic regression analysis was used for multivariate analysis and calculation of odds ratio (OR) for the development of Asp and its resolution with first‐line antibiotics. The log‐rank test was used for comparing outcomes (PFS, LRC, and OS) between the cohort of patients with and without AsP. COX regression analysis was used for the calculation of hazard ratio (HR) with Efron’s method of tie handling, with non‐AsP cohort being considered as a reference. The proportional hazard assumption was tested prior to performing the COX regression analysis. A *p* value of 0.05 was considered significant.

## 3 RESULTS

### 3.1 Incidence

**FIGURE 1:**
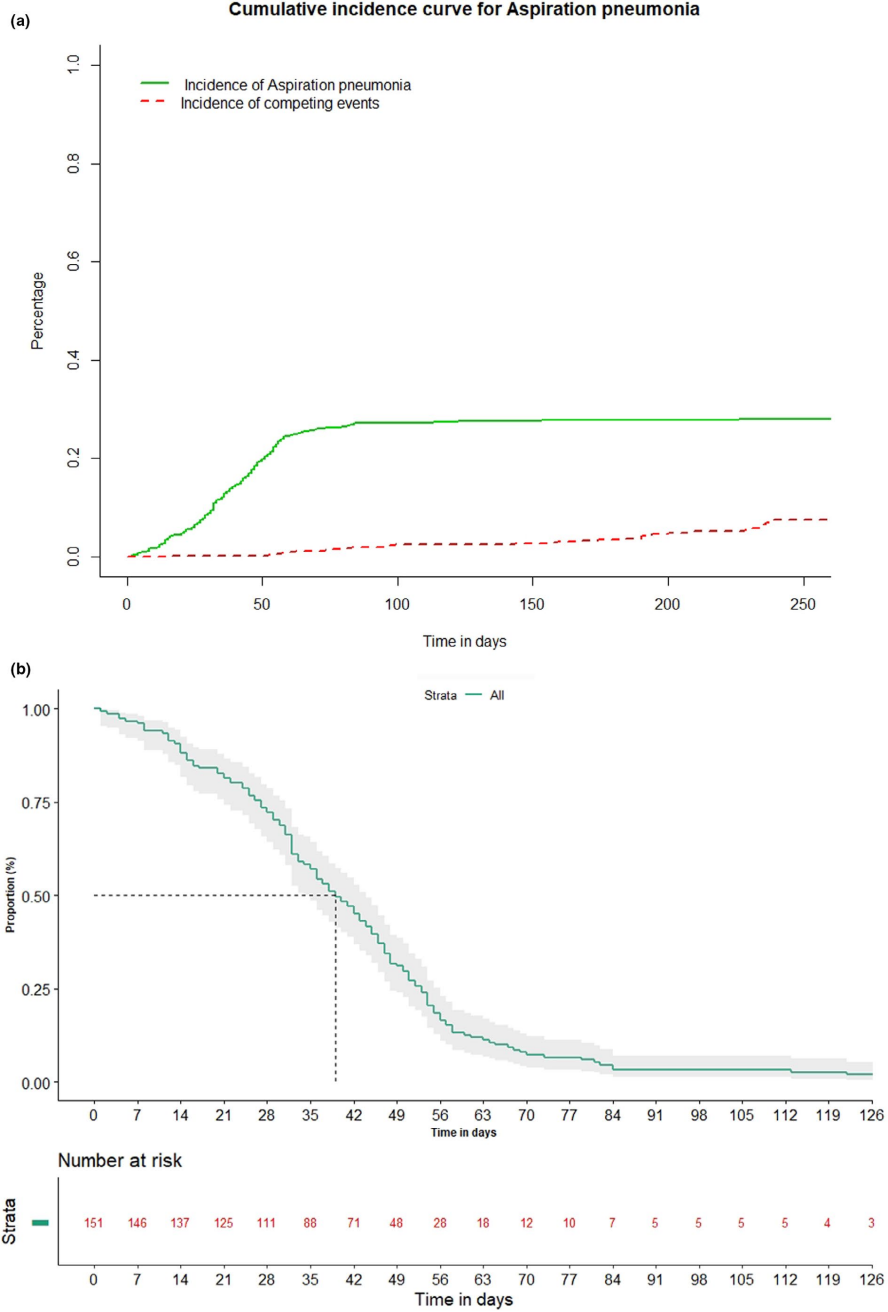
(A) Cumulative incidence curve for aspiration pneumonia (AsP). (B) Time to development of aspiration pneumonia among 151 AsP patients

Out of 536 patients enrolled in the study, 151 (28.3%, 95% CI 24.5–32.1) patients developed AsP. The cumulative incidence of development of AsP estimated by competing risk analysis was 27.99% as shown in Figure 1A. The median time to develop AsP was 39 days (95% CI 34–44) (Figure 1B). Post 49 days (7 weeks) 47 patients (29.8%, 95% CI 22.7–37.2) developed AsP.

### 3.2 Risk factors

The results of univariate analysis of risk factors for the development of AsP are shown in Table 1. Association of AsP with mucositis and weight loss was checked. The rate of Asp was 34% (109, *n* = 321) in patients with grade 3 or above mucositis, as opposed to 19.5% (42, *n* = 215) in cohorts without grade 3 or above mucositis (*p* = 0.000). AsP was observed in 33.3% (2, *n* = 6) of patients who had grade 3 or above weight loss versus 28.1% (149, *n* = 530) in those without, during chemoradiation (*p* = 0.676). As adverse event data were not available at baseline, these parameters were not included in the regression model. On binary logistic regression analysis, only baseline dysphagia (OR 3.76, 95% CI 1.05–3.51) was associated with a significant risk of development of AsP (Table 1).

**TABLE 1:** Risk factors associated with the development of aspiration pneumonia

Variable	Aspiration pneumonia cohort	No aspiration pneumonia cohort	*p* value	Odds ratio (95% CI)	*p* value on multivariate
Age, no. (%)					
Elderly (*n* = 159)	44 (27.7)	115 (72.3)	0.916	0.98 (0.64–1.5)	0.917
Non‐elderly (*n* = 377)	107 (28.4)	270 (71.6)		Reference	
Gender, no. (%)					
Male (*n* = 457)	126 (27.6)	331 (72.4)	0.498	Reference	0.672
Female (*n* = 79)	25 (31.6)	54 (68.4)		1.12 (0.65–2)	
Site, no. (%)					
Oropharynx (*n* = 269)	73 (27.1)	196 (72.9)	0.114	Reference	0.444
Larynx (*n* = 155)	38 (24.5)	117 (75.5)		1.1 (0.64–1.74)	0.841
Hypopharynx (*n* = 109)	40 (36.7)	69 (63.3)		1.5 (0.9–2.4)	0.111
Oral cavity (*n* = 3)	—	3 (100)		0	0.999
Stage, no. (%)					
III (*n* = 167)	39 (23.4)	128 (76.6)	0.098	0.69 (0.43–1.1)	0.115
IV (*n* = 369)	112 (30.4)	257 (69.6)		Reference	
Alcohol use, no. (%)					
Yes (*n* = 132)	35 (26.5)	97 (73.5)	0.657	Reference	0.815
No (*n* = 404)	116 (28.7)	288 (71.3)		1.06 (0.66–1.68)	
Pretreatment dysphagia, no(%)					
Grade 0–2	144 (27.4)	381 (72.6)	0.014	Reference	0.042
Grade 3–4	7 (63.6)	4 (36.4)		3.76 (1.05–13.51)	
Treatment, no. (%)					
CRT	68 (25.4)	200 (74.6)	0.179	0.81 (0.55–1.19)	0.283
NCRT	83 (31)	185 (69)		Reference	
Pretreatment serum albumin, no. (%)					
≥3.5 g/dl	144 (27.7)	376 (72.3)	0.166	Reference	0.379
<3.5 g/dl	7 (43.8)	9 (56.2)		1.61 (0.56–4.61)	

The percentages provided are calculated with sample size of each row.

CI, confidence interval; CRT, chemoradiation; NCRT, nimotuzumab and chemoradiation.

### 3.3 Presenting features and microbiology

Among the 151 patients with AsP, the diagnosis was based on clinical criteria in 116 patients (76.8%), radiological criteria in 2 patients (1.3%), and on both criteria in 33 patients (21.9%). Desaturation with room air was seen in 16 (10.6%) patients and hypotension was seen in 14 patients (9.3%). Sputum culture was available in 110 patients (72.8%, *n* = 151). Among them, a pathogenic organism was isolated in 69 patients (45.7%, *n* = 151). One pathogenic organism was isolated in 45 patients (29.8%), 2 in 19 (12.6%) patients, and 3 in 5 (3.3%) patients. Gram‐negative organisms were seen in 63 (89%, 95% CI 81.9–96.2) out of 69 patients. The microbial details with culture sensitivity spectrum are shown in Table 2.

**TABLE 2:** Table depicting the microorganism antibiotic sensitivity pattern

Variables	*Klebsiella pneumonia*	*Pseudomonas* aeruginosa	*Acinetobacter baumannii*	*Staphylococcus aureus*	*Escherichia coli*	*Shewanella putrefaciens*	*Enterobacter cloacae*	*Streptococcus pneumoniae*	*Enterobacter aerogenes*
Number of isolates	29	28	7	7	7	5	4	3	2
Ceftazidime	27 (93.1)	25 (89.3)	4 (57.1)	0	7 (100)	5 (100)	4 (100)	0	2 (100)
Cefotaxime	22 (75.9)	1 (3.6)	2 (28.6)	0	3 (42.9)	0	4 (100)	3 (100)	2 (100)
Ciprofloxacin	27 (93.1)	26 (92.9)	5 (71.4)	2 (28.6)	1 (14.3)	5 (100)	4 (100)	0	2 (100)
Amikacin	29 (100)	27 (96.4)	1 (100)	0	7 (100)	5 (100)	3 (75)	0	2 (100)
Cefoperazone–sulbactam	29 (100)	25 (89.3)	4 (57.1)	0	6 (85.7)	5 (100)	4 (100)	0	2 (100)
Piperacillin/Tazobactam	27 (93.1)	25 (89.3)	4 (57.1)	0	6 (85.7)	5 (100)	4 (100)	0	1 (50)
Gentamicin	26 (89.7)	26 (92.9)	4 (57.1)	5 (71.4)	7 (100)	5 (100)	4 (100)	0	2 (100)
Cefazolin	18 (62.1)	0	0	3 (42.9)	1 (14.3)	0	0	0	0
Tobramycin	7 (24.1)	26 (92.9)	5 (71.4)	0	0	5 (100)	3 (75)	0	1 (50)
Vancomycin	0	0	0	7 (100)	0	0	0	3 (100)	0
Teicoplanin	0	0	0	7 (100)	0	0	0	3 (100)	0
Linezolid	0	0	0	7 (100)	0	0	0	3 (100)	0
Erythromycin	0	0	0	4 (57.1)	0	0	0	1 (33.3)	0
Clindamycin	0	0	0	3 (42.9)	0	0	0	1 (33.3)	0
Penicillin	0	0	0	7 (100)	0	0	0	0	0

### 3.4 Treatment details

**FIGURE 2:**
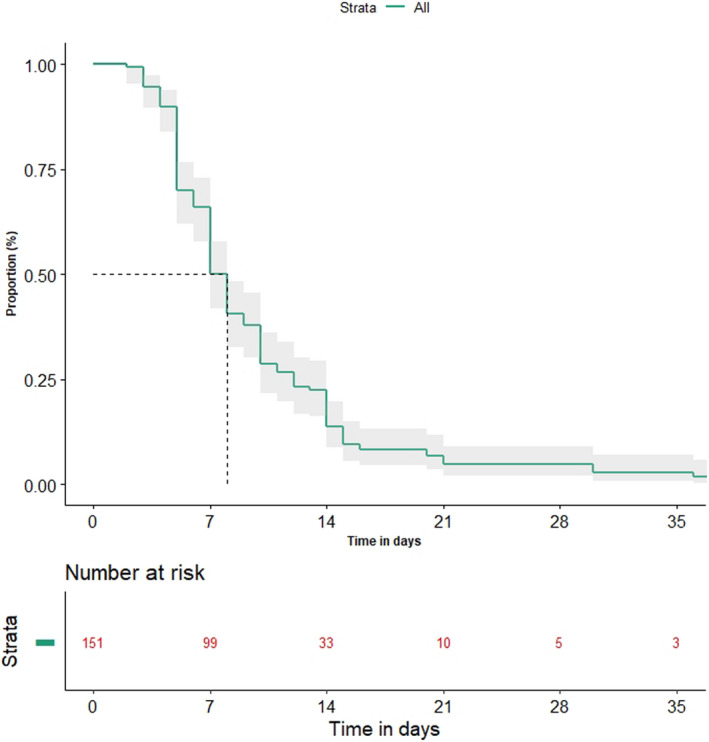
Figure depicting time to resolution of symptoms of aspiration pneumonia,

All 151 patients received first‐line antibiotics. Broad‐spectrum antibiotics were received in 101 patients (66.9%) (Table 3). Response to first‐line antibiotics was seen in 131 patients (80.1%). Most patients were started on broad‐spectrum antibiotics in first‐line setting like cefoperazone–sulbactam (45%), amoxycillin–clavulanic acid (22.5%), teicoplanin (7.9%), and meropenem (1.3%) as shown in Table 3. Combination antibiotics were used in 71 (47%) patients at first line and 3 (15%) patients at second line. In 20 patients (19.9%) a change in antibiotics to second line was required. The most common second‐line antibiotics used were cefoperazone–sulbactam (75%), teicoplanin (15%), meropenem (15%), amikacin (5%), and colistin (5%). The median time to recovery was 8 days (95% CI 7–8 days) (Figure 2). The factors predicting hazard of longer recovery are shown in Table 4. Intensive care visits were required in five patients (3.1%) and there were five deaths due to AsP (3.1%). These deaths due to AsP were sudden and occurred within 48 h of presentation.

**TABLE 3:** Table depicting the antibiotic usage pattern

Variable	Value (%)
First‐line antibiotics (*n* = 151)	
Cefoperazone–sulbactam	68 (45)
Amoxycillin–clavulanic acid	34 (22.5)
Azithromycin	8 (5.3)
Ceftriaxone	9 (6.0)
Cefuroxime	7 (4.6)
Levofloxacin	53 (35.1)
Ciprofloxacin	20 (13.2)
Linezolid	4 (2.6)
Clindamycin	5 (3.3)
Teicoplanin	12 (7.9)
Meropenem	2 (1.3)
Subsequent antibiotics (*n* = 20)	
Cefoperazone–sulbactam	15 (75)
Amikacin	1 (5)
Teicoplanin	3 (15)
Colistin	1 (5)
Meropenem	3 (15)

The % value for first‐line antibiotics is calculated with *n* = 151 while for the second line using *n* = 20. The percentages does not add up to 100 as combination antibiotics were used in 71 (47%) patients at first line and 3 (15%) patients at second line.

**TABLE 4:** Table showing factors affecting the resolution of symptoms on first‐line antibiotics

Variable	Variable division	Recovery on first‐line antibiotic number (%)	Odds ratio	*p* value
Age	Elderly (*n* = 44)	40 (90.9)	Reference	0.653
	Non‐elderly (*n* = 107)	91 (85)	0.755 (0.221–2.574)	
Diabetes mellitus	Present (*n* = 11)	10 (90.9)	Reference	0.349
	Absent (*n* = 140)	121 (86.4)	0.324 (0.031–3.428)	
Severity of pneumonia	Severe (*n* = 27)	19 (70.4)	Reference	0.004
	Non‐severe (*n* = 124)	112 (90.3)	5.433 (1.725–17.11)	
Use of broad‐spectrum antibiotics	No (*n* = 50)	38 (76)	Reference	0.006
	Yes (*n* = 101)	93 (92.1)	4.329 (1.524–12.346)	

### 3.5 Impact on cancer direct therapy

There was no difference in radiation dose received between the two cohorts. Planned radiation dose was completed in 144 (95.4%) patients and 358 (93%) patients in AsP and non‐AsP cohorts, respectively (*p* = 0.209). There was a difference in the cisplatin dose received between the two cohorts. A cumulative cisplatin dose of 200 mg/m^2^ or above was received by 312 (81%) patients in the non‐AsP cohort versus 107 (70.9%) patients in AsP cohort, respectively (*p* = 0.014). This was due to a lower number of cisplatin cycles received by the AsP cohort. Seven or more cisplatin cycles were received by 111 (73.5%) patients in the AsP cohort as opposed to 334 (86.8%) patients in the non‐AsP cohort (*p* = 0.000).

### 3.6 Impact on cancer outcomes

**FIGURE 3:**
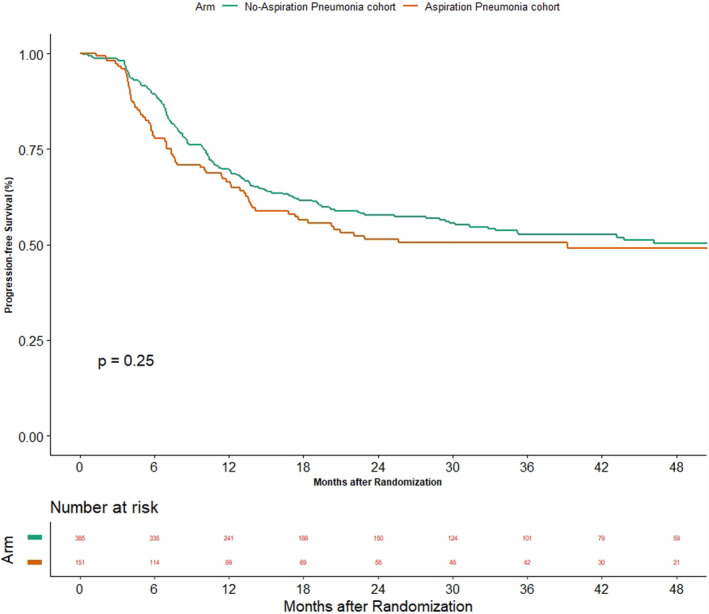
Figure depicting the impact of aspiration pneumonia on progression‐free survival

**FIGURE 4:**
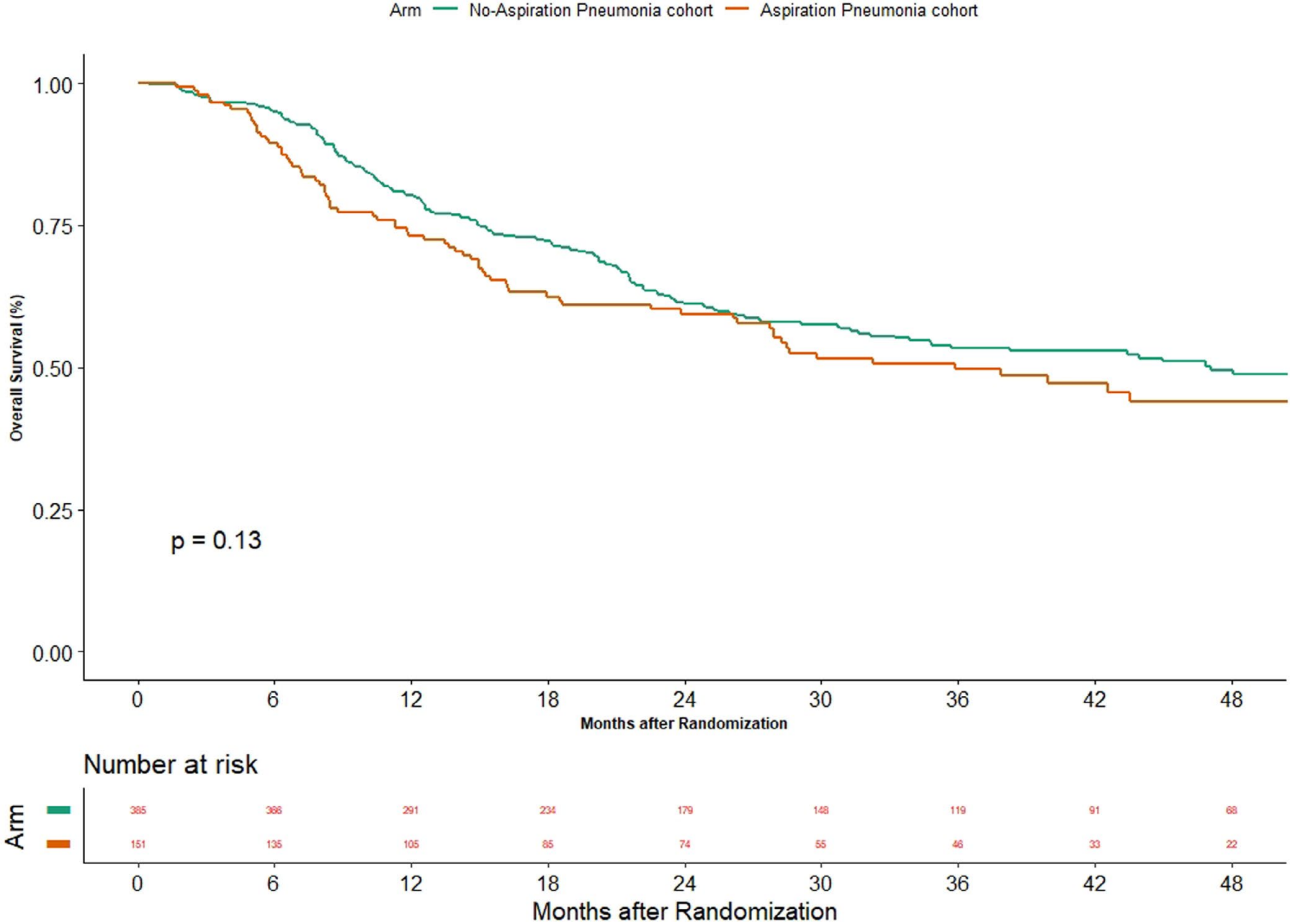
Figure depicting the impact of aspiration pneumonia on overall survival

The oncological outcomes between the two groups are shown in Figures 3, 4 and 3, 4. The median PFS was 39.2 months (95% CI 16.8–NA) versus 56.3 (95% CI 29.7–NA) in AsP and non‐AsP cohorts, respectively (*p* = 0.25). The HR for progression was 1.176 (95% CI 0.89–1.553). The median time to locoregional failure was 60.3 months (95% CI 39.2–NA) versus not reached (95% CI 56.3–NA) in AsP and non‐AsP cohorts, respectively (*p* = 0.73). The HR for locoregional failure was 1.057 (95% CI 0.771–1.448). The median OS in the cohort of patients with AsP was 35.9 months (95% CI 26.3–60.3) while it was 47.1 months (95% CI 32.1–NA) (*p* = 0.13) in non‐AsP cohort. The HR for death was 1.233 (95% CI 0.939–1.618).

## 4 DISCUSSION

Aspiration pneumonia can occur during any time post start of therapy in head and neck cancer. In this study, we focused on acute pneumonia which occurred within 180 days of completion of chemoradiation. This, to our knowledge, is the first comprehensive data on acute AsP in head and neck cancer patients undergoing CTRT from an LMIC. The audit revealed some interesting and clinically useful data. The incidence of AsP was 28.3%. The incidence of acute AsP within 1 year of treatment, in retrospective series, is between 5.3% and 15.8%.^7^, ^11^, ^12^ Our incidence of AsP seems higher than that reported in these series. The reason could be that all patients in this study had undergone video fluoroscopic examination and those with known aspiration were watched closely for the development of pneumonia, thus leading to better detection. Higher detection of rate of AsP is possible if patients are prospectively observed for the development of features of pneumonia. In a phase 3 randomized study evaluating the impact of prophylactic antibiotics on the development of AsP, the incidence of AsP observed was 46.8%^6^ showing that it was not uncommon.

Risk factors for AsP are female gender, pretreatment dysphagia, hypopharyngeal primary, advanced stage, and use of alcohol and baseline serum albumin.^3^, ^13^, ^16^, ^17^ Most of these risk factors either predispose to aspiration or are associated with poor dental hygiene or malnourishment. Hence we tested for these factors. However, in our large series, only pretreatment dysphagia grade 3–4 was an independent risk factor with >3 times the odds of developing AsP (Table 1). Factors related to malnutrition and poor dental hygiene did not have an impact on AsP in our series. This highlights the importance of the baseline supportive care and corrective measures provided by the speech–swallowing expert, dentist, and the nutritionist which probably negated the impact of these features.

Patients were assessed weekly during chemoradiation. However, in spite of weekly assessments, nearly 10% of patients presented with either hypotension and /or desaturation highlighting the sudden and aggressive nature of this complication. Hence, prompt attention to the assessment of aspiration and symptoms of pneumonia during a weekly review during chemoradiation is critical. The median time to develop AsP was 39 days that is 5–6th week. This is the time when most patients have grade 3 and above mucositis, making them vulnerable to aspiration. Hence, assessing patients with severe mucositis for the features of aspiration is essential. Nearly one third patients develop AsP after completion of chemoradiation that is after 7 weeks. So, prior to discharge, it is important to assess the swallowing function and keep patients having aspiration under close surveillance. Furthermore, those with baseline dysphagia need to be educated about the signs and symptoms of AsP and the importance of immediate treatment.

In 45% of patients, a pathogen was isolated, with 14.6% of patients having more than one pathogen identified. The spectrum of pathogens was mainly gram‐negative organisms (>80%) with resistance to penicillins (Table 2). The type of organisms and the resistant patterns suggest that broad‐spectrum antibiotic coverage inclusive of gram‐negative bacteria coverage should be considered in these patients. In our analysis, the use of broad‐spectrum antibiotics was associated with a higher resolution rate (Table 4) underlining the importance to have an institutional antibiotic policy in patients undergoing chemoradiation especially those at risk of AsP. These data differs significantly with AsP data from high‐income countries where gram‐positive bacteria (>60%) would be the predominant causative agent.^18^, ^19^ AsP‐related mortality was seen in only 3.3% of patients in the current series, which is much lower than that reported in retrospective series (30%).^7^, ^12^ Mortality similar to ours was reported in the European randomized study evaluating the role of prophylactic antibiotics in AsP.^6^ Early anticipation in at‐risk patients and prompt antibiotic therapy can substantially decrease mortality.

There was a trend toward inferior outcome in AsP cohort but it was not statistically significant. The impact on the development of AsP on treatment outcomes seen in our series is not in line with the results of some retrospective series.^3^, ^7^, ^10^, ^20^ The reason was probably due to immediate and prompt initiation of broad spectrum antibiotics as soon as aspiration pneumonia was suspected in our patients. The clinicians involved in the study did not halt radiation due to AsP unless the patient was unstable. However, concurrent systemic therapy was withheld in few patients till recovery of symptoms as per the severity of pneumonia.

The data in the study were from a single center. This may question the generalizability of the data across other LMICs. However, the study was conducted in Tata Memorial Centre which sees around >10,000 new cases of head and neck cancer per year.^21^ These are contributed by multiple states in and around the city of Mumbai. The geographical size of many of these states is larger than most nations in Europe, thus suggesting that the data are generalizable. We have not performed axial imaging in most of the patients. This actually helps to provide pragmatic data that can be applied across most LMICs, where a chest x‐ray alone is routinely performed. We have restricted our analysis to acute AsP as we aimed to understand the importance of this adverse event during, and immediately after chemoradiation.

The strength of the this study is that this is probably the first study on Asp from an LMIC providing substantial detailed data on incidence, treatment, and its impact on oncological outcomes. The data of AsP were prospectively collected. The cases were labeled as AsP based on strict criteria and sputum cultures were performed in >70% of patients with pathogens identified in 61.8% of patients in whom culture was performed.

## 5 CONCLUSION

Aspiration pneumonia is a common adverse event in head and neck cancer management and patients with baseline dysphagia are at high risk. Gram negative bacteria are the predominant causative agents in LMIC. The use of broad‐spectrum antibiotics as the first line is likely to result in resolution of symptoms and recovery. Aggressive management of AsP is associated with low mortality and minimal impact on treatment outcomes.

## ETHICS STATEMENT

The study was approved by the Institutional Ethics Committee and was conducted in accordance with the Declaration of Helsinki and the International Committee on Harmonization of Good Clinical Practice (ICH‐GCP). The study was registered with the clinical trial registry of India (CTRI).

## CONFLICT OF INTEREST

Vanita Noronha reports grants from Dr. Reddy's Laboratories, Amgen, and Sanofi, outside of the submitted work. Kumar Prabhash reports grants from Tata Memorial Center Research Administration Council, the Indian Cooperative Oncology Network, and Glenmark Pharmaceuticals, during the conduct of the study; grants from Dr. Reddy's Laboratories, Fresenius Kabi India, Alkem Laboratories, Natco Pharma, BDR Pharmaceuticals India, and Roche Holding, outside of the submitted work. All other authors declare no competing interests.

## Data Availability

All authors consent that data used for analysis will be made available to Cancer Medicine Journal on request.

## References

[1] Ferlay J , Soerjomataram I , Dikshit R , et al. Cancer incidence and mortality worldwide: sources, methods and major patterns in GLOBOCAN 2012. Int J Cancer. 2015;136:E359‐E386. 10.1002/ijc.29210. 25220842

[2] Sankaranarayanan R . Oral cancer in India: an epidemiologic and clinical review. Oral Surg Oral Med Oral Pathol. 1990;69:325‐330. 2179801 10.1016/0030-4220(90)90294-3

[3] Shirasu H , Yokota T , Hamauchi S , et al. Risk factors for aspiration pneumonia during concurrent chemoradiotherapy or bio‐radiotherapy for head and neck cancer. BMC Cancer. 2020;20:182. 32131771 10.1186/s12885-020-6682-1 PMC7057640

[4] Madan R , Kairo AK , Sharma A , et al. Aspiration pneumonia related deaths in head and neck cancer patients: a retrospective analysis of risk factors from a tertiary care centre in North India. J Laryngol Otol. 2015;129:710‐714. 26077504 10.1017/S0022215115001450

[5] Kanayama N , Otozai S , Yoshii T , et al. Death unrelated to cancer and death from aspiration pneumonia after definitive radiotherapy for head and neck cancer. Radiother Oncol. 2020;151:266‐272. 32866561 10.1016/j.radonc.2020.08.015

[6] Ham JC , Driessen CM , Hendriks MP , et al. Prophylactic antibiotics reduce hospitalisations and cost in locally advanced head and neck cancer patients treated with chemoradiotherapy: a randomised phase 2 study. Eur J Cancer. 2019;113:32‐40. 30965213 10.1016/j.ejca.2019.02.013

[7] Xu B , Boero IJ , Hwang L , et al. Aspiration pneumonia after concurrent chemoradiotherapy for head and neck cancer. Cancer. 2015;121:1303‐1311. 25537836 10.1002/cncr.29207 PMC4774546

[8] Ward MC , Adelstein DJ , Bhateja P , et al. Severe late dysphagia and cause of death after concurrent chemoradiation for larynx cancer in patients eligible for RTOG 91–11. Oral Oncol. 2016;57:21‐26. 27208840 10.1016/j.oraloncology.2016.03.014

[9] Lindblom U , Nilsson P , Gärskog O , et al. Aspiration as a late complication after accelerated versus conventional radiotherapy in patients with head and neck cancer. Acta Otolaryngol. 2016;136:304‐311. 26838580 10.3109/00016489.2015.1113439

[10] Kawai S , Yokota T , Onozawa Y , et al. Risk factors for aspiration pneumonia after definitive chemoradiotherapy or bio‐radiotherapy for locally advanced head and neck cancer: a monocentric case control study. BMC Cancer. 2017;17:59. 28095814 10.1186/s12885-017-3052-8 PMC5241959

[11] Hutcheson KA , Nurgalieva Z , Zhao H , et al. Two‐year prevalence of dysphagia and related outcomes in head and neck cancer survivors: an updated SEER‐Medicare analysis. Head Neck. 2019;41:479‐487. 30536748 10.1002/hed.25412 PMC6355350

[12] Mortensen HR , Jensen K , Grau C . Aspiration pneumonia in patients treated with radiotherapy for head and neck cancer. Acta Oncol. 2013;52:270‐276. 23173758 10.3109/0284186X.2012.742205

[13] Kawashita Y , Morimoto S , Tashiro K , et al. Risk factors associated with the development of aspiration pneumonia in patients receiving radiotherapy for head and neck cancer: retrospective study. Head Neck. 2020;42:2571‐2580. 32478453 10.1002/hed.26314

[14] Goyal G , Patil VM , Noronha V , et al. Once‐a‐week versus once‐every‐3‐weeks cisplatin in patients receiving chemoradiation for locally advanced head‐and‐neck cancer: a survey of practice in India. Cancer Res Stat Treat. 2018;1:63‐67.

[15] Patil VM , Noronha V , Joshi A , et al. A randomized phase 3 trial comparing nimotuzumab plus cisplatin chemoradiotherapy versus cisplatin chemoradiotherapy alone in locally advanced head and neck cancer. Cancer. 2019;125(18):3184‐3197. 31150120 10.1002/cncr.32179

[16] Reddy PD , Yan F , Nguyen SA , Nathan C‐AO . Factors Influencing the development of pneumonia in patients with head and neck cancer: a meta‐analysis. Otolaryngol Head Neck Surg. 2021;164(2):234‐243. 32660345 10.1177/0194599820938011

[17] Purkey MT , Levine MS , Prendes B , Norman MF , Mirza N . Predictors of aspiration pneumonia following radiotherapy for head and neck cancer. Ann Otol Rhinol Laryngol. 2009;118:811‐816. 19999368

[18] Polverino E , Dambrava P , Cillóniz C , et al. Nursing home‐acquired pneumonia: a 10 year single‐centre experience. Thorax. 2010;65:354‐359. 20388763 10.1136/thx.2009.124776

[19] Carratalà J , Mykietiuk A , Fernández‐Sabé N , et al. Health care‐associated pneumonia requiring hospital admission: epidemiology, antibiotic therapy, and clinical outcomes. Arch Intern Med. 2007;167:1393‐1399. 17620533 10.1001/archinte.167.13.1393

[20] Chen S‐W , Yang S‐N , Liang J‐A , Lin F‐J . The outcome and prognostic factors in patients with aspiration pneumonia during concurrent chemoradiotherapy for head and neck cancer. Eur J Cancer Care. 2010;19:631‐635. 10.1111/j.1365-2354.2009.01104.x 20109165

[21] User, S. Accessed January 12, 2021. https://tmc.gov.in/index.php/en/tmc‐annual‐report

